# Electric stimulation of ears accelerates body weight loss mediated by high-fat to low-fat diet switch accompanied by increased white adipose tissue browning in C57BL/6 J mice

**DOI:** 10.1186/s12906-018-2388-1

**Published:** 2018-12-05

**Authors:** Szu-Han Chen, Hsiao-Chien Chen, Ching-Liang Hsieh, Pei-Min Chao

**Affiliations:** 1 Institute of Nutrition, China Medical University, Taichung, 40402 Taiwan; 2 Department of Nutrition, China Medical University, Taichung, 40402 Taiwan; 3 Graduate Institute of Integrated Medicine, College of Chinese Medicine, China Medical University, Taichung, 40402 Taiwan; 4 Chinese Medicine Research Center, China Medical University, Taichung, 40402 Taiwan; 5 Department of Chinese Medicine, China Medical University Hospital, Taichung, 40447 Taiwan

**Keywords:** Auricular electric stimulation, Dietary control, Anti-obesity, UCP-1, Browning of white adipose tissue

## Abstract

**Background:**

Weight reduction frequently occurs in patients receiving vagus nerve stimulation (VNS) therapy. Therefore, we hypothesized that during dietary intervention for weight loss, auricular electric stimulation (AES), an alternative of VNS, accelerates weight loss by increasing white adipose tissue (WAT) browning and increases energy expenditure.

**Methods:**

C57BL/6J male mice were fed a high-fat diet for 5 wk. to induce obesity, then switched to a low-fat diet for 5 wk. and allocated into 3 groups to receive 2 Hz electric stimulation on ears, electrode clamps only, or nothing (AES, Sham and Ctrl, respectively).

**Results:**

Switching to a low-fat diet reduced body weight progressively in all 3 groups, with the greatest reduction in the AES group. In accordance with a mild decrease in feed intake, hypothalamus mRNA levels of *Npy*, *AgRP* tended to be reduced, while *Pomc* tended to be increased by AES. Mice in the AES group had the highest concentrations of norepinephrine in serum and inguinal WAT, and expression levels of uncoupling protein-1 (UCP-1) and tyrosine hydroxylase in inguinal WAT. Furthermore, their subcutaneous adipocytes had multilocular and UCP-1^+^ characteristics, along with a smaller cell size.

**Conclusion:**

AES, by increasing WAT browning, could be used in conjunction with a low-fat diet to augment weight loss in addition to suppressing appetite.

## Background

Obesity is a worldwide epidemic and is causally linked with many chronic diseases [1]. To tackle this problem, it should be noted that obesity is a complex and multi-factorial disease which involves the integration of social, cultural, behavioral, physiological, neuroendocrinal, and genetic factors. For many people, adherence to the principle of weight reduction, namely eating less and exercising more, is difficult. Bariatric surgery and pharmacological treatment are only applicable for severe or morbid obesity. Therefore, there is a strong impetus to develop alternative approaches.

Electrical stimulation of the vagus nerve (VNS), approved by the Food and Drug Administration (FDA) for treating refractory epilepsy and resistance depression, is known to cause weight loss [2–6]. Ear skin (locating in concha) has the greatest density of branches of the vagus nerves derived from the superior jugular ganglion of the vagus [7]. Therefore, application of auricular VNS or acupuncture in weight reduction is common, an effect mainly attributable to the suppressed appetite [8–11]. Recently, auricular VNS was reported to increase brown adipose tissue (BAT) activity [12, 13]. BAT is highly sympathetic innervated, with thermogenic functions triggered by norepinephrine released from sympathetic nerve terminals and mediated by a β3-adrenoceptor/cAMP-activated protein kinase pathway [14].

White and brown are 2 distinct adipose tissues for storage of excess energy and thermogenesis, respectively. In addition to traditional white and brown adipocytes, a third type of adipocyte, i.e. brown-in-white (brite) or beige cells, that emerge within white adipose tissue (WAT) are regarded as a plastic response to an energy surplus [14]. Brite adipocytes evolve from a different lineage than brown adipocytes [14]; they are inducible, multilocular, and express a BAT-specific uncoupling protein-1 (UCP-1) marker for thermogenesis [15]. UCP-1, also called thermogenin, resides in the mitochondrial inner membrane and uncouples the respiratory chain from oxidative phosphorylation by disrupting the H^+^ gradient across the membrane. Contributions of BAT to anti-obesity were largely ignored until functional BAT in adult humans was identified by ^18^fluorodeoxyglucose-positron emission tomography-computed tomography (FDG-PET-CT) in supraclavicular and neck regions, with molecular signatures that resembled murine brite rather than classical brown adipocytes present in interscapular BAT [16–18]. Furthermore, human BAT activity was inversely correlated with adiposity [16, 19]. Recruiting more brite cells or increasing WAT browning by pharmacological or transgenic approaches turn out to be a fascinating strategy for anti-obesity since it improved glucose tolerance and anti-steatosis as well [20].

There existed an anatomical relationship between the auricular branch of the vagus nerve and the nucleus tractus solitarius (NTS), the primary vagal afferent center [21, 22]. Electric stimulation at exterior margin of the auricle, i.e. auricular electric stimulation (AES), has the same auricular VNS effects, i.e. increasing firing rate of NTS neurons and parasympathetic tone [23–25]. The exterior margin of the auricle is mainly innervated by the great auricular nerve (GAN) and the central projections of the GAN to NTS has been demonstrated by neural tracing study [26]. Accordingly, AES can be used as an alternative of auricular VNS. In this study, we hypothesized that during dietary intervention for weight loss, AES accelerates weight loss by increasing WAT browning. The weight reduction effect was tested in diet-induced obese (DIO) mice switched to a standard chow diet with or without AES. Histological and molecular markers for WAT browning were checked in subcutaneous inguinal WAT as it is most susceptible to browning among all fat depots [27].

## Methods

### Animals and diets

**Fig. 1:**
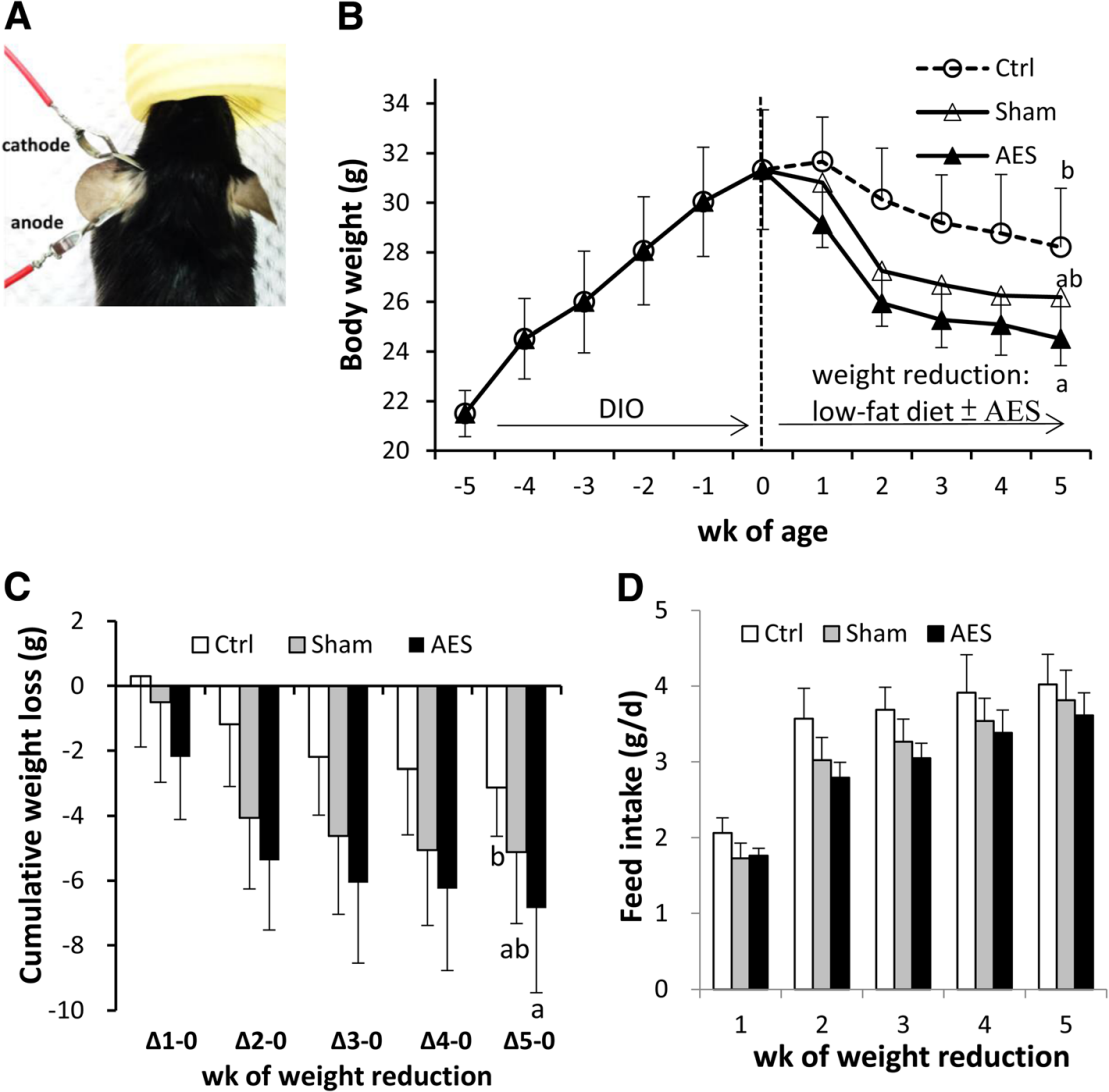
Application of clip electrodes on mouse ears (**a**), body weight throughout the study (**b**), as well as cumulative weight loss (**c**) and daily feed intake (**d**) during low-fat diet intervention period of mice in three groups. Data are mean ± SD, *n* = 8. ^a,b^Means without a common letter differed (*P* < 0.05)

Twenty four male C57BL/6JNarl mice were purchased from the National Laboratory Animal Center of the National Applied Research Laboratories, Taipei, Taiwan. At 6 wk. of age, all mice were fed a butter-based high-fat diet (30% dietary fat comprised of 29% butter and 1% soybean oil) for 5 wk. to induce obesity [indicated as wk. − 5〜0 in Fig. 1b]. C57BL/6 J mouse is prone to obesity and diabetes in response to a high-fat diet [28, 29]. The diet composition as reported in Chen et al. [30] has been verified to induce obesity and associated metabolic disorders in mice [30, 31]. Their body weight reached 31.3 ± 2.4 g at wk0 in contrast to 24.0 ± 1.6 g of chow-diet fed peers (> 20% higher). After DIO, mice were weighed and allocated by body weight into 3 groups (*n* = 8 for each group), i.e. AES (+/+ for electricity/clamps), Sham (−/+ for electricity/clamps), and Ctrl (−/− for electricity/clamps). They were fed a low-fat non-purified diet (Altromin 1320 Rat & Mouse Maintenance diet, Fwusow Industry Co. Ltd., Taiwan; containing 6% water, 51% crude carbohydrate, 23.5% crude protein, 4.5% crude lipid, 6% crude fiber, and 9% ash) onward for 5 wk. and concomitantly, AES or sham operation were applied during this period [indicated as wk. 0〜5 in Fig. 1b]. The body weight of Ctrl, Sham and AES group at wk. 0 was not different (i.e. 30.8 ± 2.6, 31.3 ± 2.4 and 31.5 ± 1.8 g, respectively). All mice were housed in polypropylene cages in groups of four mice per cage and were kept in a room maintained at 23 ± 2 °C, with a controlled 12-h-light:-dark cycle with ad libitum access to feed and drinking water. Body weight and feed intake were recorded twice per week. Cumulative body weight loss (from baseline, i.e. switching point of diet) was calculated each week. Animal care and research protocols were based on principles and guidelines approved by the Guide for the Care and Use of Laboratory Animals [32]. Protocols for animal care and handling were approved by the Institutional Animal Care and Use Committee of China Medical University (Protocol 103–69-N).

### Auricular electrostimulation

AES was applied under anesthesia by isoflurane administered through a vaporizing system (MATRX VIP 3000, Midmark, USA). Mice in the AES group received electrical stimulation (frequency, 2 Hz; intensity, 2 mA; visual ear twitch) using clip electrodes (ES apparatus Trio 300, Ito, Japan) with the anode placed at the ear lobe and cathode at the ear apex [23–25], as shown in Fig. 1a. Stimulation was done 20 min/d (with each ear receiving the stimulus for 10 min) and 3 d each week (13:00–16:00 on Monday, Wednesday, and Friday) for 5 wk. consecutively. For the Sham group, anesthesia and clip electrodes were applied but no electricity was given and Ctrl mice were only anesthetized.

### Tissue sampling and biochemical analysis

At the end of the study, feed was withheld overnight and the mice were killed by carbon dioxide asphyxiation. Blood was collected from the abdominal vena cava, allowed to clot and serum separated. WAT (mesenteric, retroperitoneal, epididymal, and inguinal fat) were excised and weighed. Aliquots of inguinal fat and hypothalamus were quick-frozen in liquid nitrogen and stored at − 80 °C for RNA or protein extraction. Concentrations of catecholamines (epinephrine and norepinephrine) in serum and inguinal fat (RIPA buffer extract) were measured using commercial kits (R&D, Minneapolis, MN), following manufacturer’s instructions.

### RNA isolation and mRNA detection

The mRNA levels of *Npy, AgRP* and *Pomc* (encoding neuropeptide Y, agouti-related peptide and pro-opiomelanocortin, markers for appetite regulation) in hypothalamus, as well as *Ucp1* (markers for WAT browning) in inguinal fat were measured by qRT-PCR. Total RNA was extracted from homogenized tissue using TRIZOL reagent (Invitrogen, Carlsbad, CA, USA) according to the manufacturer’s instructions and 1 mg total RNA was reverse-transcribed into first-strand cDNA using 200 units of MMLV-RT (Promega, Madison, WI, USA) in a total volume of 20 mL. For real-time PCR, a TaqMan system with inventory primers and probes (Applied Biosystems, Foster City, CA, USA) or a SYBR system with self-designed primers was used (Table 1). Amplification using 40 cycles of 2 steps (95 °C for 15 s and 60 °C for 1 min) was done with an ABI Prism 7900HT sequence detection system.

**Table 1:** Assay ID of the inventory primers and probes and the sequence of the self-designed primers used for qRT-PCR

Gene	Accession number	Assay ID or primer sequence
*Ucp1*	NM_009463.3	Mm01244861_m^1^
*Npy*	NM_023456.3	F: CAGAACAAGGCTTGAAGACC C R: GCAGACTGGTTTCAGGGGAT
*AgRP*	NM_007427.3	F: GAGTTCCCAGGTCTAAGTCTGAATG R: ATCTAGCACCTCCGCCAAAG
*Pomc*	NM_008895	F: CCCGCCCAAGGACAAGCGTT R: CTGGGCCCTTCTTGTGCGCGT

^1^Inventory primers and probes purchased from Applied Biosystems

### Immunoblotting

Samples of inguinal fat (0.1 g) were homogenized in RIPA buffer which contained 1% protease inhibitor cocktail (Sigma, St. Louis, MO, USA). Appropriate amounts of homogenate containing 50 mg of protein were electrophoresed on 10% SDS gels, transferred to a PVDF transfer membrane, and immunoblotted. Primary antibodies used were mouse antibodies against β-actin (St John’s Laboratory, London, UK), tyrosine hydroxylase (TH; Millipore, California) and rabbit antibodies against human UCP-1 (Abcam, Cambridge, UK) (diluted 1:1000 in PBS). In addition, HRP-labeled goat anti-mouse IgG antibodies (Jackson ImmunoResearch, West Grove, PA, USA) and goat anti-rabbit IgG antibodies (Abcam) at a dilution of 1:5000 in PBS were used as a secondary antibody. Bound antibodies were detected using an enhanced chemiluminescence Western blotting kit (Amersham International, Uppsala, Sweden) and images quantified by densitometric analysis (Multimage Light Cabinet, Alpha Innotech Corporation, San Leandro, CA, USA).

### Immunohistochemical analyses

A portion of inguinal fat was fixed in 10% formalin, dehydrated through a graded ethanol series, embedded in paraffin, and cut into 5 μm sections. Sections were incubated with 5% goat serum in PBS after deparaffinization and rehydration. The primary antibody was a rabbit antibody against human UCP-1 (Abcam) (diluted 1:100 in PBS), whereas the secondary antibody was biotinylated goat anti-rabbit IgG antibodies (Dako, Carpinteria, CA, USA) (diluted 1:250 in PBS). Sections for UCP-1 staining were processed using a Dako kit (Dako REALTM envision TM detection system) according to the manufacturer’s instructions and examined on a Primo Star microscope (Zeiss, Oberkochen, Germany). Adiposoft software (ImageJ; National Institutes of Health, Bethesda, MD, USA) was used to calculate adipocyte cell diameter.

### Statistical analyses

Data were expressed as mean ± SD. Comparisons among groups were done with 1-way ANOVA and Duncan’s multiple range test. If variances were not homogeneous, data were log-transformed prior to analysis. The General Linear Model (SAS, SAS Institute, Cary, NC, USA) was used for statistical analyses and differences were considered significant at *P* < 0.05.

## Results

### Effects of AES on weight reduction

As expected, body weight was linearly increased by a high-fat diet, whereas switching to a low-fat diet caused a rapid decrease in body weight at the 2nd wk., followed by less rapid declines, irrespective of AES or not (Fig. 1b). However, when body weight change (Δ) were calculated and expressed as cumulative weight loss from baseline (switching point of diet), there was a trend of AES > Sham>Ctrl across the 5-wk weight reduction period, with a difference (AES vs. Ctrl, *P* < 0.05) at the last week (Fig. 1c). Although daily feed intake was not significantly different among groups, there was an opposite trend to weight loss, i.e. AES < Sham<Ctrl (Fig. 1d).

There were no significant differences among groups for body fat percentage in mesenteric, retroperitoneal, epididymal, and inguinal fat pads (Table 2**)**. However, adipocyte diameter in inguinal fat (representative of subcutaneous fat) in the AES group was significantly smaller than in Ctrl, with an intermediate value for Sham (Table 2).

**Table 2:** Body fat (%) and adipocyte diameter (mm)^1^

	Mesenteric fat	Retroperitoneal fat	Epididymal fat	Inguinal fat	Adipocyte diameter^2^
Ctrl	0.07 ± 0.03	0.06 ± 0.01	0.28 ± 0.06	0.31 ± 0.1	0.19 ± 0.02^a^
Sham	0.06 ± 0.04	0.05 ± 0.02	0.23 ± 0.04	0.27 ± 0.07	0.17 ± 0.02^a^
AES	0.08 ± 0.06	0.05 ± 0.03	0.25 ± 0.05	0.30 ± 0.11	0.12 ± 0.01^b^

^1^Values are mean ± SD, *n* = 8. ^a,b^ Within a column, means without a common superscript differ (*P*<0.05)

^2^Measured in inguinal fat

### Effects of AES on catecholamines and appetite

**Fig. 2:**
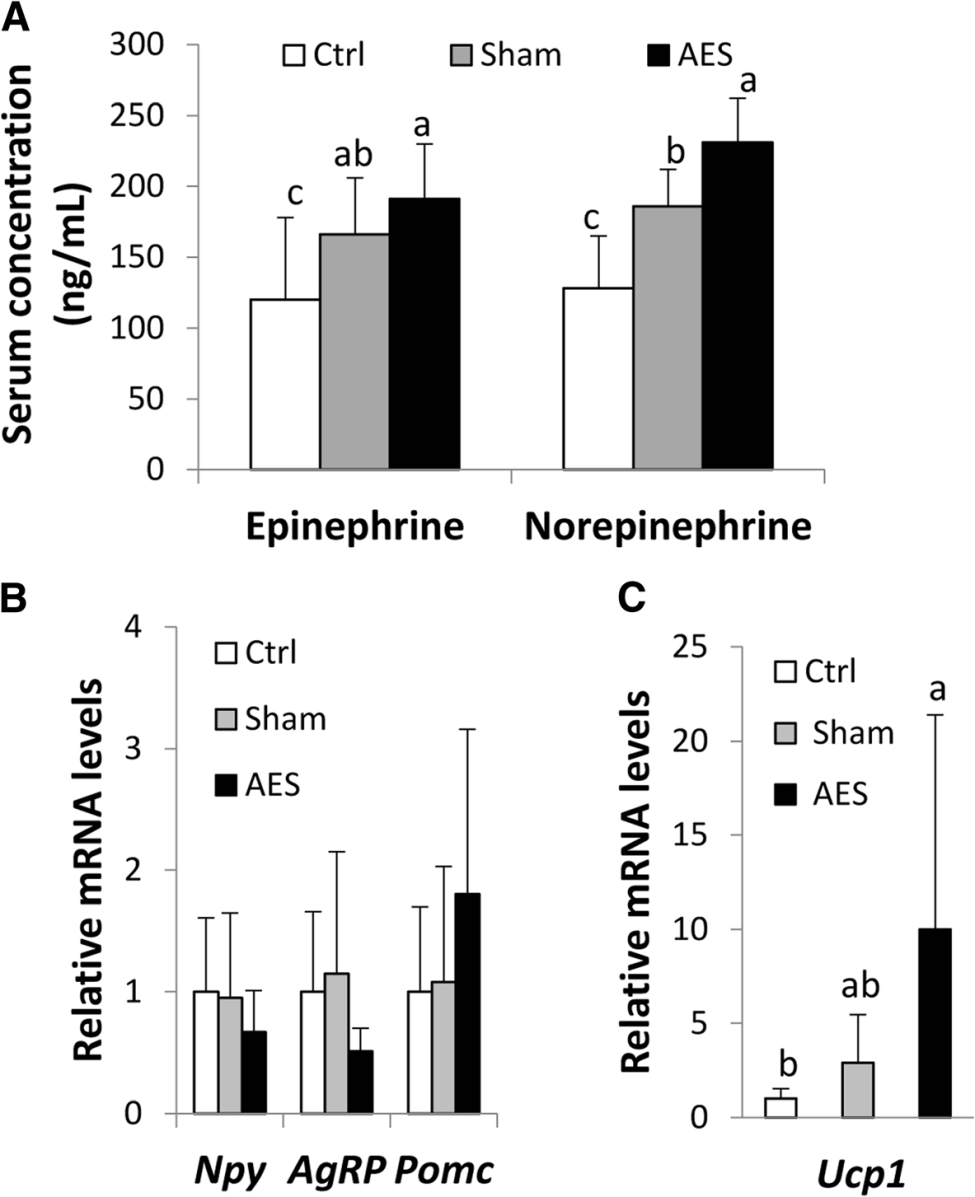
Serum catecholamine concentrations (**a**) and mRNA levels of markers associated with appetite regulation in hypothalamus (**b**) and WAT browning in inguinal fat (**c**) of mice in three groups. Data are mean ± SD, n = 8. In (**b**) and (**c**), the comparison was based on the mRNA levels relative to Ctrl (taking as 1). ^a-c^Means without a common letter differed (*P* < 0.05)

Serum concentrations of epinephrine and norepinephrine were elevated by auricular stimulation, regardless of whether electricity was applied (Fig. 2a), although concentrations were highest in the AES group. For norepinephrine, concentrations in AES were significantly greater than Sham, and the values in both groups were significantly greater than that of Ctrl.

Hypothalamic transcripts for neuropeptides associated with appetite regulation did not differ among groups, though the orexigenic *Npy* and *AgRP* tended to be lowered, while anorexigenic *Pomc* tended to be elevated in the AES group (Fig. 2b).

### Effect of AES on WAT browning

**Fig. 3:**
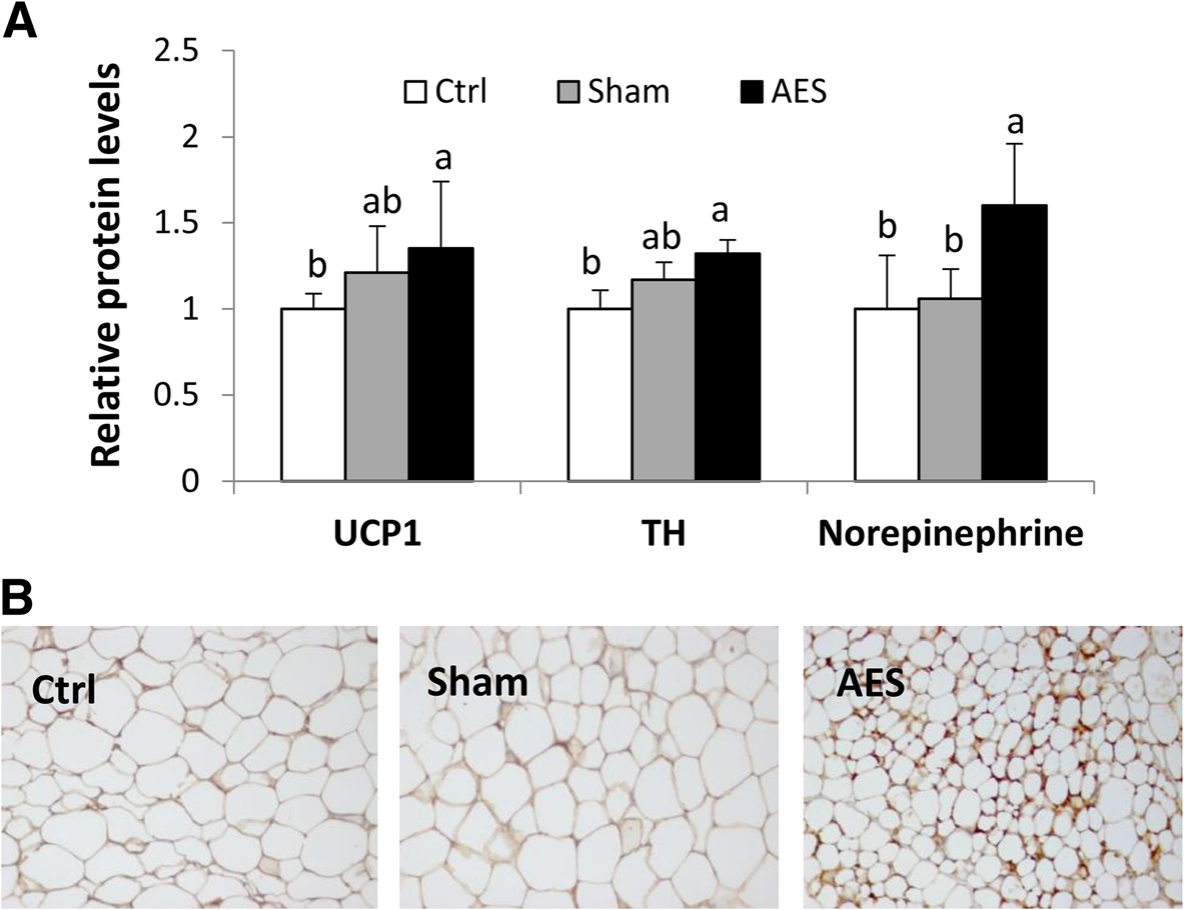
Protein levels of markers associated with thermogenesis and sympathetic innervation (**a**) and IHC staining for UCP-1 (**b**) in inguinal WAT of mice in three groups. In (**a**), Data are mean ± SD, n = 8. In (**b**), the comparison was based on the protein levels relative to Ctrl (taking as 1). ^a,b^Means without a common letter differed (*P* < 0.05)

The mRNA levels of *Ucp1* in inguinal fat were significantly greater in group AES than Ctrl, with an intermediate value for Sham (Fig. 2c). Protein levels of UCP-1 and TH in inguinal fat were greatest for AES, followed by Sham, and lowest for Ctrl (AES vs. Ctrl, *P* < 0.05; Fig. 3a). Norepinephrine concentrations in inguinal fat did not differ between groups Sham and Ctrl, with both significantly lower than in AES. In the latter group, there was morphological and immunohistochemical evidence of WAT browning, with adipocytes with multilocular and UCP-1^+^ staining characteristics (Fig. 3b), along with a smaller cell size relative to the other 2 groups.

## Discussion

In weight reduction programs, a major challenge is that caloric restriction results in negative energy balance, triggering an adaptive response of reducing metabolic rate and energy expenditure, thereby making it difficult to lose weight. Therefore, any strategy that can mitigate this adaptive response, for example exercise or AES, would be beneficial. In the present study, a low-fat diet intervention resulted in progressive weight loss; the addition of AES further accelerated weight loss, along with increased subcutaneous WAT browning to augment energy expenditure. There was a slight but non-significant effect in sham-operated mice, attributed to physical stress. The mRNA and protein levels of UCP-1, in addition to histologic evidence (multilocular with UCP-1^+^), confirmed that there were more brite cells in the AES group.

AES, via VNS, increases parasympathetic activity [25, 33]. Furthermore, postganglionic parasympathetic nerves release predominantly acetylcholine. It is well established that BAT has abundant sympathetic noradrenergic nerves [14]. Although cholinergic nerve fibers were present in mediastinal BAT, its relevance to thermogenesis (a catabolic process) remains unknown [34]. Based on responses of antagonists of muscarinic acetylcholine receptors, it appeared that parasympathetic nerves had an inhibitory role in thermogenesis in obese rats [35]. Kreier et al. reported the presence of a parasympathetic input in WAT, which played a stimulating anabolic role following fat pad–specific vagotomy [36]. This was not consistent with AES activating BAT; however, we inferred that the increased britening in WAT in the AES group was through norepinephrine-mediated sympathetic activity, rather than parasympathetic activation (discussed below).

There are two reports regarding VNS impact on BAT thermogenic activity. Vijgen et al. recruited 15 epilepsy patients on stable VNS therapy and conducted BAT activity measurement by FDG-PET-CT [12]. Despite the lack of a significant difference in BAT activity between active and inactive VNS therapy, energy expenditure decreased significantly when VNS was inactivated, with the change in energy expenditure significantly related to changes in BAT activity. In another study, application of auricular VNS to high-fat fed Sprague-Dawley rats enhanced reductions in adiposity, and was accompanied by higher serum norepinephrine concentrations and greater expression of β_3_-adrenoceptor and UCP-1 in the BAT [13]. We are the first to report that AES resulted in WAT browning. It is believed that norepinephrine released from the sympathetic nerve endings in BAT or WAT binds to β_3_-adrenoceptors on adipocytes and initiates intracellular G-coupled protein signaling (cAMP-activated protein kinase), leading to intracellular breakdown of triglycerides to provide free fatty acids for thermogenesis via UCP-1. The shrinkage of adipocytes observed in inguinal WAT of AES group supports this note (Table 2).

It remains elusive how VNS increased sympathetic norepinephrine innervation on BAT or WAT. From an anatomic perspective, the vagus nerve mediates peripheral signals and relays these information to NTS in the brainstem and then projects them to the central nervous system, putatively working through interactions among the hypothalamic arcuate nucleus, paraventricular nucleus and ventromedial nucleus, which in turn connects to sympathetic nerves that innervate tissue expressing adrenoceptors [21]. The link between parasympathetic vagal signaling and central sympathetic was supported by the finding that subdiaphragmatic vagotomy impaired the BAT-mediated diet-induced thermic response [37]. Acute VNS increased norepinephrine concentrations and its transmission in rat brain [38, 39], which has a role in VNS therapeutic efficacy for epilepsy and depression [38, 40]. The link between the parasympathetic and sympathetic system was also evidenced in inflammatory control [41].

Significantly higher serum norepinephrine concentration in the AES group was consistent with a previous study [13]. Furthermore, AES increased norepinephrine concentrations in subcutaneous WAT. In accordance with this, tyrosine hydroxylase protein concentration was also increased by AES in inguinal fat. This enzyme is responsible for catecholamine synthesis and is commonly used as a marker for sympathetic innervation in adipose tissue [42]. The current results supported the assertion that AES increased sympathetic innervation in subcutaneous WAT, although the mechanism of action is yet to be established. It was reported that VNS or cholinergic agonists are able to activate the noradrenergic splenic nerve [41]. The AES-mediated elevation in serum norepinephrine came from adrenal or other sources in addition to WAT awaits for further studies. Moreover, it will be interesting to test the balance of autonomic nervous system (sympathetic or parasympathetic predominance) in this scenario.

Interestingly, either VNS with 0.01 to 30 Hz or vagal blockade (VBLOC) with reversible vagal inhibition achieved by applying kilohertz frequency current directly to the nerve to block localized electrical conduction, was effective in treating obesity [8]. The former was attributed to increase satiation, reduced sweet cravings, and increased energy expenditure [43], whereas the latter was attributed to preventing aberrant orexigenic vagal afferent signaling in obesity [44]. There are many reports that VNS prevented excessive weight gain and suppressed food intake in high-fat diet-fed animals [13, 44–46]. However, the present study was apparently the first to test auricular VNS in combination with low-fat diet treatment on DIO mice. In line with a slightly lowered feed intake, the hypothalamic mRNA levels of orexigenic *Npy* and *AgRP* tended to be lowered and anorexigenic POMC tended to be elevated by AES. This was in line with the role of vagus nerve in gut–brain signaling [8]. Ear acupuncture stimulation was demonstrated to exert a sympathomimetic effect that temporarily increased basal metabolic rate and decreased appetite [47]. Therefore, satiation, more than WAT browning, may also have contributed to weight reduction.

One limitation of this study is there was no fat pad weight difference was observed, perhaps a 5 wk-intervention was too short for this to be manifest. In that regard, a significant increase of AES on cumulative weight loss was not detected until the last (5th) wk. intervention, indicating more prolonged intervention may be needed to reveal its impact on body fat mass. In addition, dissected fat-pad weights might not be sensitive enough to show the mild changes. Whole body composition (adiposity, lean body mass, and body water) measured by nuclear magnetic resonance would be more informative. Nevertheless, 5 wk. of AES treatment suppressed hypertrophic adipocytes within subcutaneous WAT and cell diameter in the AES group was reduced by 40% of that in the Ctrl group.

To eliminate the interference of gender, only male mice were used in this study as they are prone to DIO than females. For future perspectives, it is intriguing to test the sex effect on weight reduction of AES. In future clinical trials, metabolic examinations (such as serum glucose, free fatty acids, cholesterol…etc) as well as autonomic balance between sympathetic and parasympathetic tone (such as heart rate and heart rate variability of R-R intervals) should be incorporated to explain the possible mechanisms of AES action.

## Conclusions

In this study, AES accelerated weight loss induced by a low-fat diet, suggesting that it could be used as an ancillary method to enhance weight loss, perhaps in combination with dietary/behavioral modifications. The underlying mechanisms were regarded as increased energy expenditure from WAT browning, in addition to a mild suppression on appetite. The advantage to using AES in weight reduction programs is it relatively safe compared to pharmacotherapy, which has numerous side effects. Moreover, it is less invasive compared to FDA approved VNS or VBLOC. Though further investigation on its dose/duration and impacts on energy homeostasis are required, AES has potential for use in weight management.

## Abbreviations

AES: auricular electric stimulation
BAT: Brown adipose tissue
DIO: Diet-induced obese;
FDA: The Food and Drug Administration
FDG-PET-CT: ^18^fluorodeoxyglucose-positron emission tomography-computed tomography
TH: Tyrosine hydroxylase
UCP-1: Uncoupling protein-1
VBLOC: Vagal blockade
VNS: Vagus nerve stimulation
WAT: White adipose tissue
